# Beyond the Trinity of ATM, ATR, and DNA-PK: Multiple Kinases Shape the DNA Damage Response in Concert With RNA Metabolism

**DOI:** 10.3389/fmolb.2019.00061

**Published:** 2019-08-02

**Authors:** Kaspar Burger, Ruth F. Ketley, Monika Gullerova

**Affiliations:** Sir William Dunn School of Pathology, University of Oxford, Oxford, United Kingdom

**Keywords:** DNA damage response, RNA metabolism, phosphorylation, RNA polymerase II, dicer, kinase, 53BP1

## Abstract

Our genome is constantly exposed to endogenous and exogenous sources of DNA damage resulting in various alterations of the genetic code. DNA double-strand breaks (DSBs) are considered one of the most cytotoxic lesions. Several types of repair pathways act to repair DNA damage and maintain genome stability. In the canonical DNA damage response (DDR) DSBs are recognized by the sensing kinases Ataxia-telangiectasia mutated (ATM), Ataxia-telangiectasia and Rad3-related (ATR), and DNA-dependent protein kinase (DNA-PK), which initiate a cascade of kinase-dependent amplification steps known as DSB signaling. Recent evidence suggests that efficient recognition and repair of DSBs relies on the transcription and processing of non-coding (nc)RNA molecules by RNA polymerase II (RNAPII) and the RNA interference (RNAi) factors Drosha and Dicer. Multiple kinases influence the phosphorylation status of both the RNAPII carboxy-terminal domain (CTD) and Dicer in order to regulate RNA-dependent DSBs repair. The importance of kinase signaling and RNA processing in the DDR is highlighted by the regulation of p53-binding protein (53BP1), a key regulator of DSB repair pathway choice between homologous recombination (HR) and non-homologous end joining (NHEJ). Additionally, emerging evidence suggests that RNA metabolic enzymes also play a role in the repair of other types of DNA damage, including the DDR to ultraviolet radiation (UVR). RNAi factors are also substrates for mitogen-activated protein kinase (MAPK) signaling and mediate the turnover of ncRNA during nucleotide excision repair (NER) in response to UVR. Here, we review kinase-dependent phosphorylation events on RNAPII, Drosha and Dicer, and 53BP1 that modulate the key steps of the DDR to DSBs and UVR, suggesting an intimate link between the DDR and RNA metabolism.

## Introduction

Chromosomes encode essential genetic information that needs to be faithfully inherited by daughter cells to maintain genome stability and prevent tumourigenesis. However, numerous exogenous and endogenous factors such as ionizing radiation (IR), ultraviolet radiation (UVR), pathogens, reactive oxygen species, or chemotherapeutic drugs can frequently induce DNA damage. If such lesions are not repaired correctly they have the potential to drive mutations of genes, leading to detrimental effects on genomic integrity. Various lesion-specific pathways repair damaged DNA by a signaling network collectively termed the DNA damage response (DDR) to maintain genome stability and prevent alterations of genomic information (Jackson and Bartek, 2009).

DNA double-strand breaks (DSBs) are highly cytotoxic forms of DNA damage, which impair essential cellular processes including DNA replication and RNA synthesis, which if left unrepaired, can lead to cell death. However, various physiological processes such as meiosis (de Massy, 2013), V(D)J recombination, and immunoglobulin class-switch recombination (Soulas-Sprauel et al., 2007), also inherently involve formation, recognition and repair of DSBs. Mammalian cells employ two major types of DSB repair in the context of chromatin and the cell cycle: homologous recombination (HR) and non-homologous end joining (NHEJ) (Jackson and Bartek, 2009; Ciccia and Elledge, 2010; Polo and Jackson, 2011; Chapman et al., 2012; Her and Bunting, 2018; Hnízda and Blundell, 2019).

HR can be utilized in the S-/G_2_-phase of the cell cycle, after a sister chromatid template with sufficient homology of >100 base pairs has been produced in replication. HR also requires extensive resection of DNA ends, which generates 3' single-stranded DNA overhangs and engages factors such as exonuclease Exo1, the single-strand DNA-binding protein RPA, and the Rad51 recombinase. Unlike HR, NHEJ requires no nucleotide homology and is active throughout the cell cycle. However, the precision of NHEJ repair is lower than in HR and can lead to mutagenesis. Important NHEJ factors include DNA end-binding heterodimer Ku70/80, the DNA ligase 4, DNA endonuclease Artemis/SNM1C and XRCC4. Interestingly, HR and NHEJ pathways compete for the DSB substrate in the S-/G_2_-phase. Around 80% of DSBs are repaired by NHEJ, despite the second chromatid being available (Chapman et al., 2012; Pannunzio et al., 2018). Additional types of DSB repair include microhomology-mediated end joining (MMEJ), and single-strand annealing (SSA) (Verma and Greenberg, 2016; Chang et al., 2017).

Three key phosphatidylinositol 3-kinase-related kinase (PIKK) family members, Ataxia Telangiectasia mutated (ATM), Ataxia Telangiectasia and Rad3-related (ATR), and DNA-dependent protein kinase (DNA-PK) orchestrate the DDR by phosphorylating hundreds of substrates (Kastan and Lim, 2000; Matsuoka et al., 2007; Blackford and Jackson, 2017). ATR and DNA-PK sense DSBs in cooperation with the Mre11-Rad50-Nbs1 (MRN) complex leading to the activation of downstream kinases such as checkpoint kinase 1/2 (Chk1/2) (Blackford and Jackson, 2017).

Downstream of the sensing kinases, 53BP1 is a key factor in DNA double strand break (DSB) repair, regulating repair pathway choice between HR and NHEJ. 53BP1 promotes NHEJ and represses HR by preventing DNA end resection at DSBs through antagonism with BRCA1 (Shibata, 2017). 53BP1 recruitment to DSBs begins following MRN recruitment to DSBs, whereupon ATM is recruited and phosphorylates histone H2AX on Ser139 (γH2AX) (Panier and Boulton, 2014; Mirza-Aghazadeh-Attari et al., 2019).

Additionally, several kinases beyond PIKKs act in the DDR. Intriguingly, about 40% of DSB-induced phosphorylation events occur independent of ATM and may regulate processes related to nucleic acid metabolism, including RNA processing and chromatin organization (Bennetzen et al., 2010; Bensimon et al., 2010). In fact, the majority of DSB-induced phospho-proteins indeed lack a PIKK consensus motif (Beli et al., 2012). These findings not only suggest an important role for downstream kinases as amplifiers of DSB signaling, but also establish regulatory links between the DDR and RNA metabolic enzymes. Moreover, the interplay between the DDR and RNA metabolic enzymes is not limited to the recognition and repair of DSBs. Some of the regulatory principles for DSB-induced regulation of the RNA metabolism are mirrored in response to UV damage. Here, we review recent advances in our understanding of the regulatory phosphorylation events that control the RNA-dependent DDR. We illustrate their relevance for genome stability by describing the various types of 53BP1 engagement in DSB repair. We will further compare similarities and differences of RNAi factors involved in DSB repair with their contribution to the recognition and repair of UV lesions.

## Global Repression and Local Induction of Transcription in Response to DSBs

Unscheduled or excessive transcription is generally regarded as a threat to genome stability. Therefore, RNA synthesis is tightly controlled and coordinated with DNA replication timing to avoid collisions of the transcription and replication machineries, otherwise leading to replication fork collapse and accumulation of DNA-RNA hybrids (R-loops). Whilst R-loops can form as intermediates in certain cellular processes such as IgG class switch recombination and transcription (Skourti-Stathaki and Proudfoot, 2014; Skourti-Stathaki et al., 2014), formation of R-loops exposes single-stranded, non-template DNA strand, which can lead to an increase in mutagenesis, DNA breaks, and subsequent formation of DSBs (Huertas and Aguilera, 2003; Skourti-Stathaki and Proudfoot, 2014; Hamperl and Cimprich, 2016; Hamperl et al., 2017). RNAPII transcription of protein-coding genes is globally impaired in response to DSBs. Onset of ATM signaling triggers the damage-induced ubiquitination of RNAPII by Nedd4 ubiquitin ligase and its subsequent proteasomal degradation (Anindya et al., 2007; Shanbhag et al., 2010). Combination of site-specific induction of DSBs by the AsiSI endonuclease and sequencing of both steady-state and nascent RNA revealed that ATM-dependent downregulation of RNAPII at protein-coding genes occurs at the level of RNAPII initiation and elongation and also depends on the distance from the DSBs (Iannelli et al., 2017). Similarly, DNA-PK arrests elongating RNAPII at DSBs within protein-coding genes (Pankotai et al., 2012).

The recognition and repair of DSBs is accompanied by substantial changes in the chromatin landscape to allow DNA repair by HR and/or NHEJ pathways. Various chromatin-modifying enzymes and remodeling machineries such as PBAF facilitate silencing of actively transcribed loci by formation of non-permissive heterochromatin (Kakarougkas et al., 2014). Furthermore, ATM-dependent phosphorylation of the transcription elongation factor ENL facilitates recruitment of the polycomb repressor complex to silence transcription (Ui et al., 2015). Finally, the Cohesin complex, well-known for sister chromatid cohesion and DSBs repair through HR, is required for repression of transcription in damaged interphase nuclei and the organization of DSBs into higher-order chromatin structures (Meisenberg et al., 2019). Thus, an active, damage-induced transcriptional response associated with DSBs might seem surprising.

**Figure 1:**
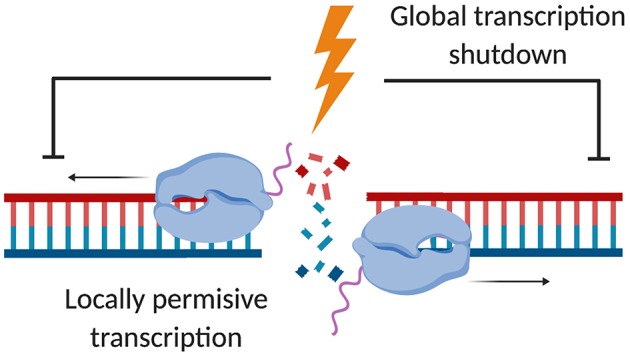
Locally permissive transcription in response to DSBs despite global transcription shutdown. Upon DSB induction, RNAPII transcription of protein-coding genes is globally impaired via ATM signaling. Non-permissive heterochromatin forms to facilitate the silencing of actively transcribed loci. However, transcriptionally permissive open chromatin has been suggested to form locally in response to DSBs, allowing nascent RNA synthesis to occur at the site of the DSB (created by Biorender).

Early *in vitro* studies suggest that RNAPII can transcribe linearised plasmids by recognizing DNA ends with 10–100 nts 3′overhangs (Kadesch and Chamberlin, 1982). The ability of RNAPII to transcribe RNA from linearised plasmids can also be observed in cells, where RNAPII components are part of the DNA end-binding proteome (Michalik et al., 2012; Berthelot et al., 2016). Surprisingly, physiological DSBs promote gene expression *in vivo*. A subset of early synaptic response genes is induced upon DNA damage by inhibition of topoisomerase II in neurons (Madabhushi et al., 2015). Additionally, stimulation of RNAPII activity by androgens or estrogens involves formation of DSBs, also mediated by topoisomerase II (Haffner et al., 2011). The stimulation of RNAPII elongation involves components of DSB signaling such as DNA-PK and topoisomerase II (Bunch et al., 2015). Intriguingly, DSBs are repaired faster if they occur at actively transcribed loci with transcriptionally active chromatin directing DSB repair toward the HR pathway (Chaurasia et al., 2013; Aymard et al., 2014). Data utilizing the sequence-specific AsiSI cleavage demonstrate that histone marks associated with active transcription, such as histone H4 acetylation, accumulate at a subset of AsiSI induced DSBs. Furthermore, RNAPII occupancy correlates with nucleosome-free regions rather than being disengaged from AsiSI-restricted chromatin (Iacovoni et al., 2010). More recently, systematic profiling of epigenetic marks in response to AsiSI cleavage has defined the histone H3 lysine120 (H3K120) ubiquitination mark as DSB-responsive molecular identifier of damaged DNA. H3K120 deubiquitination and acetylation depends on the SAGA multi-enzyme complex and may promote local permissive chromatin (Clouaire et al., 2018). These findings suggest that DSBs trigger chromatin breathing, which may result in a local, transiently open chromatin state to create a “window of opportunity” for transcription factors and nascent RNA synthesis (Price and D'Andrea, 2013). Indeed, the 55 kD large isoform of the major RNAPII transcription-regulating cyclin-dependent kinase 9 (Cdk9 55k), associates with the DNA end-binding Ku70 protein and depletion of Cdk9 55k induces accumulation of DSBs (Liu et al., 2010), further implying a close link between RNAPII transcription and genome stability. Given that the chromatin state impacts on genome stability—with poorly transcribed, heterochromatic regions driving mutation rates (Schuster-Böckler and Lehner, 2012)—it has been tempting to postulate that localized induction of RNA synthesis may have benefits for DSB repair. Indeed, increasing evidence suggests that an RNA-dependent response to DSB may involve the *de novo* production of strand-specific, long non-coding (lnc)RNA precursors. Such transcripts may originate from RNAPII activity at both genic and intergenic DSBs, as well as at damaged ribosomal DNA (*rDNA*) genes (Michelini et al., 2017; Bonath et al., 2018; Burger et al., 2019; Vítor et al., 2019). Damage-induced lncRNA are prone to form hybrids, such as R-loops and/or double-stranded (ds)RNA, and undergo subsequent processing by RNAi factors Drosha and/or Dicer, but may also utilize alternative enzymes for trimming and clearance (Ohle et al., 2016; Burger et al., 2017; Burger and Gullerova, 2018; D'Alessandro et al., 2018; Lu et al., 2018; Yasuhara et al., 2018). Findings in *S. cerevisiae* suggest a model of RNA-templated DSB repair, which employs both exogenous RNA oligonucleotides and endogenous lncRNA as complimentary templates for DSB repair by HR (Storici et al., 2007; Keskin et al., 2014). Similarly, nascent RNA forms a complex with actively transcribing RNAPII and a subset of NHEJ factors to mediate error-free repair of DSBs (Chakraborty et al., 2016). DSB can further utilize pre-existing or damage-induced lncRNA to scaffold recruitment of DDR factors or modulate the activity of the p53 tumor suppressor (Huarte et al., 2010; Sharma and Misteli, 2013; Schmidts et al., 2016). In summary, the relevance of RNA in DSB repair is an emerging concept, where various modes of DDR signaling may coexist to modulate transcription at DSBs, depending on the chromatin landscape and the cell cycle stage (Chowdhury et al., 2013; Michelini et al., 2018; Figure 1).

## Damage-Induced Kinases Regulate RNA Metabolism in Response to DSBs

The formation and processing of dsRNA is a consequence of damage-induced ncRNA synthesis and essential for efficient, RNA-dependent repair of DSBs. But how does DDR signaling fine-tune transcription and RNA processing at DNA lesions? Mounting evidence suggests that the DDR engages phospho-specific isoforms of RNAPII and Dicer for DSB repair by activation of damage-induced kinases, which often are activated downstream of canonical PIKK signaling (Blackford and Jackson, 2017). We and others have recently shown that the RNA-dependent response to DSBs is initiated by active RNAPII transcription. The recruitment and activity of RNAPII at broken DNA ends not only involves PIKK signaling and the MRN complex, but also hyperphosphorylation of the RNAPII carboxy-terminal domain (CTD) (Napolitano et al., 2013; Michelini et al., 2017; Burger et al., 2019). The CTD is a low complexity domain of the largest RNAPII subunit, which comprises 52 repeats of the consensus heptad Tyr1-Ser2-Pro3-Thr4-Ser5-Pro6-Ser7 and undergoes dynamic, regulatory post-translational modifications, commonly referred to as “CTD code” (Zaborowska et al., 2016; Harlen and Churchman, 2017). We have shown that the Abelson kinase c-Abl is required for the accumulation of a specific, catalytically active CTD Tyr1-phosphorylated RNAPII isoform at DSBs. Both chemical and genetic inhibition of c-Abl activity impairs the formation of CTD Tyr1-phosphorylated RNAPII foci at DSBs and attenuates its activity to produce damage responsive transcripts *de novo* (Burger et al., 2019). c-Abl is a promiscuous, nuclear tyrosine kinase with multi-faceted functions in the DDR (Colicelli, 2010; Meltser et al., 2011). c-Abl is activated by IR in a DNA-PK- and ATM-dependent manner (Baskaran et al., 1997; Kharbanda et al., 1997; Shafman et al., 1997), and phosphorylates various HR factors like Rad51 (Colicelli, 2010). Interestingly, c-Abl directly phosphorylates CTD Tyr1 residues *in vitro* (Baskaran et al., 1997; Mayer et al., 2012) and interacts with RNAPII CTD *in vivo* (Burger et al., 2019), suggesting that c-Abl, at least in part, directly regulates the accumulation and activity of CTD Tyr1-phosphorylated RNAPII at DSBs. This leads to the stepwise formation of damage-responsive transcripts and dsRNA, which are processed by the RNAi machinery and stimulate RNA-dependent recruitment of a subset of DDR factors. Intriguingly, the levels of RNAPII CTD Tyr1-phosphorylation are elevated in response to various stresses, including DNA damage, by the atypical tyrosine kinase Mpk1/Slt2 in budding yeast, whereas Mpk1/Slt2 deletion reduces, but not completely diminishes RNAPII CTD Tyr1-phosphorylation (Yurko et al., 2017). This suggests that RNAPII CTD Tyr1 phospho-marks associated with ncRNA synthesis are somewhat conserved under stress and that additional stress-responsive tyrosine kinases regulate RNAPII CTD Tyr1-phosphorylation levels. Indeed, activation of tyrosine kinase signaling is widespread during the DDR (Mahajan and Mahajan, 2015). It will be interesting to investigate additional roles for tyrosine kinases in the RNA-dependent DDR.

The requirement of Drosha and Dicer for an RNA-dependent DSB response involves formation of DSB-derived dsRNA and seems to occur independent from their canonical roles in RNAi pathway (d'Adda di Fagagna, 2014; Burger and Gullerova, 2015; Hawley et al., 2017; Pong and Gullerova, 2018). However, the molecular principles that control the formation of dsRNA or its recognition and turnover remain unclear. The endoribonuclease Dicer is a largely cytoplasmic enzyme and well-known for its canonical function in micro (mi)RNA biogenesis (Ha and Kim, 2014). During development or stimulation of growth factor signaling, however, a subset of cytoplasmic Dicer is phosphorylated by the mitogen-activated protein kinase (MAPK) signaling effector Erk1/2 in *C. elegans* and mammalian cells (Drake et al., 2014). In particular, MAPK signaling phosphorylates the two human Dicer residues Ser1728 and Ser1853–Ser1712 and Ser1836 in mouse—in the carboxy-terminal, catalytically active RNaseIII and dsRNA-binding domains of Dicer. Recent studies have confirmed the importance of these carboxy-terminal phospho-residues for Dicer localization and function in phospho-mimetic Dicer mouse models (Aryal et al., 2019). The constitutive carboxy-terminal phosphorylation of murine Dicer Ser1712 and Ser1836 residues is pathogenic and causes a hypermetabolic phenotype, which is accompanied by prominent nuclear Dicer localization, defective miRNA biogenesis, and sterility. Interestingly, we recently showed that a subset of the cytoplasmic Dicer pool translocates to the nucleus in response to DSBs to process damage-induced dsRNA on chromatin. Moreover, the localization and activity of nuclear phosphorylated Dicer requires an additional phospho-mark in the Dicer platform-PAZ connector helix residue Ser1016 (Burger et al., 2017). Dicer Ser1016 phosphorylation is induced by DSB signaling, depends on PIKK activity, and is necessary and sufficient for nuclear Dicer localization. The accumulation of nuclear, phosphorylated Dicer in response to DSB induction seems to be conserved in mammals and was confirmed in primary mouse embryonic fibroblasts that express an endogenously-tagged, full-length Dicer enzyme at physiological conditions (Burger and Gullerova, 2018).

**Figure 2:**
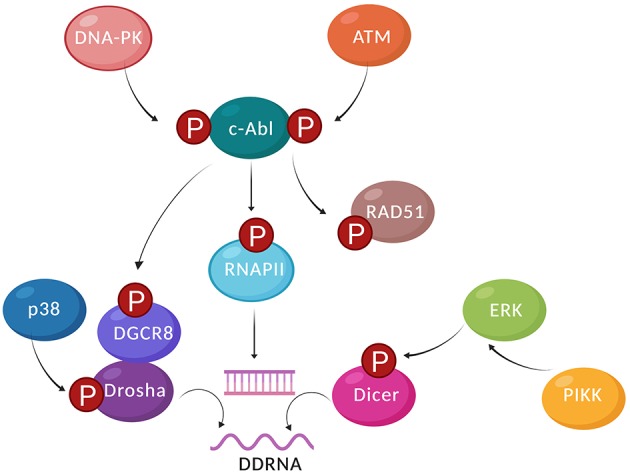
Damage-induced kinases regulate the RNA metabolism in response to DSBs. In response to DSBs, DNA-PK, and ATM phosphorylate and activate c-Abl kinase, which can phosphorylate HR factors such as Rad51. c-Abl can also phosphorylate the CTD of RNAPII at Tyr1, which is required for the recruitment and activity of RNAPII at DSBs. Damage responsive transcripts and dsRNA can then be produced at the DSB, recruiting a subset of DDR factors. Alongside RNAPII, Drosha and Dicer are also required for the formation of DSB-derived dsRNA. Cytoplasmic Dicer is phosphorylated by Erk1/2 at Ser1728 and Ser1853. Dicer phosphorylation at Ser1016 is necessary and sufficient for nuclear Dicer localization. p38 phosphorylates Drosha, promoting its dissociation from DGCR8, nuclear export of phosphorylated Drosha, and subsequent proteasomal degradation. DGCR8 can be phosphorylated by c-Abl at Tyr267, which stimulates processing of a specific miRNA precursor to promote the DDR at the post-transcriptional level (created by Biorender).

Drosha and its cofactor DiGeorge syndrome critical region 8 (DGCR8) are also subject to stress-induced post-translational modifications. The MAPK effector p38 phosphorylates the amino-terminal Arg-Ser-rich region of Drosha upon oxidative stress (Yang et al., 2015). Drosha phosphorylation promotes its dissociation from DGCR8, causes nuclear export of phosphorylated Drosha and subsequent proteasomal degradation. Stress-induced Drosha phosphorylation alters miRNA biogenesis and causes hypersensitivity to hydrogen peroxide treatment. Alternative splicing variants of Drosha localize to the cytoplasm and do not seem to alter miRNA biogenesis severely (Dai et al., 2016; Link et al., 2016). In unperturbed cells, the glycogen synthase kinase 3 beta (GSK3β) phosphorylates the two Drosha residues Ser300 and Ser302 to facilitate nuclear accumulation of Drosha and promote primary miRNA processing. Upon infection with RNA viruses, which causes pleiotropic DNA damage (Weitzman and Weitzman, 2014), a substantial amount of nuclear Drosha functions as antiviral factor. Drosha translocates to the cytoplasm to interfere with the viral RNA metabolism by sponging viral RNA. The nuclear export of Drosha is dependent on the dephosphorylation of Ser300 and Ser302 residues and is accompanied by alterations in the host transcriptome. Remarkably, Drosha interferes with viral replication independent of its catalytic activity or DGCR8 (Shapiro et al., 2014; Aguado et al., 2017). DGCR8 itself is phosphorylated by c-Abl in response to treatment with the DNA-damaging agents doxorubicin or cisplatin (Tu et al., 2015). c-Abl targets the DGCR8 residue Tyr267, which stimulates processing of a specific miRNA precursor to promote the DDR at the post-transcriptional level. Thus, various forms of cellular stress, including DNA damage, control the localization and activity of RNAi factors. It will be important to further assess the impact of DDR signaling on the post-translational modifications of RNAi factors beyond phosphorylation (Figure 2).

## RNA-Dependent and Independent Modes of 53BP1 Engagement at DSBs

53BP1 is an important regulator of DSB signaling and pathway selection between HR and NHEJ at DSBs. Canonical recruitment of 53BP1 to DSBs involves recognition of broken DNA by the MRN complex, ATM activation, and γH2A.X accumulation. The γH2A.X mark mediates the recruitment of 53BP1, Mediator of DNA damage checkpoint 1 (MDC1), and the E3 ubiquitin ligases RNF8 and RNF168 to ubiquitinated H2A marks on damaged chromatin. The recruitment of 53BP1 to DSBs further involves recognition of the H4K20me2 mark and requires ATM-dependent phosphorylation of 53BP1 itself during the S-/G_2_-phase of the cell cycle. In quiescent cells, 53BP1 is also phosphorylated by the vaccinia-related kinase 1 (VRK1) in response to IR in an ATM- and p53- independent manner. Loss of VRK1 impairs 53BP1 foci formation (Sanz-García et al., 2012). Upon recruitment to damaged chromatin, 53BP1 and its effector RIF1 promote NHEJ and repress HR by preventing BRCA1 access to DSBs. To license the HR pathway, BRCA1 together with the DNA endonuclease CtIP trigger dephosphorylation of 53BP1, which repositions 53BP1 to the periphery and allows recruitment of HR factors such as BRCA1, Exo1 and RPA to the center of the DDR focus (Daley and Sung, 2014; Lee et al., 2014; Panier and Boulton, 2014; Zimmermann and de Lange, 2014). Interestingly, the occupancy of 53BP1 on damaged chromatin may also be influenced by the Dicer-dependent regulation of the histone deacetylase sirtuin 7 (SIRT7). SIRT7 controls chromatin density and thus accessibility of 53BP1 to DSBs (Vazquez et al., 2016). In unperturbed cells, Dicer tethers a fraction of SIRT7 to the cytoplasm, thereby controlling nuclear SIRT7 levels. Upon DNA damage, however, Dicer expression may be upregulated, which further retains SIRT7 in the cytoplasm and restricts its access to chromatin. Tethering of SIRT7 to the cytoplasm decreases the levels of acetylated H3 lys18, which limits chromatin decondenzation and may eventually impair the efficient recruitment of NHEJ factors like 53BP1 (Zhang et al., 2016). However, whether or not SIRT7 deacetylation and subsequent increased H3K18 acetylation enhances or impairs NHEJ is not clear. It also remains to be clarified to what extent perturbations in miRNA biogenesis influence Dicer's contribution to the chromatin status.

**Figure 3:**
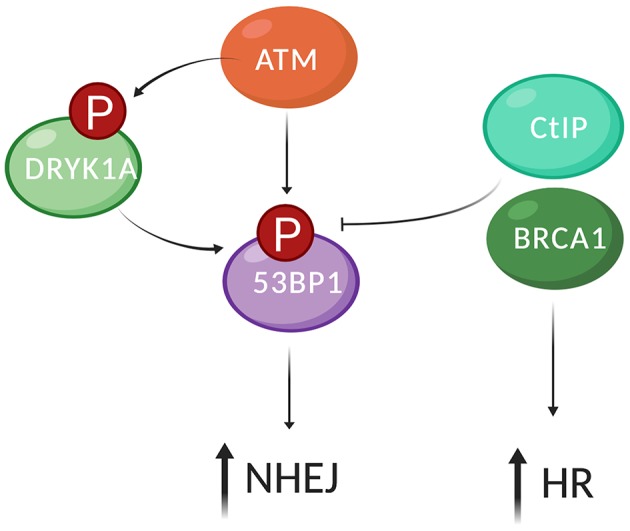
53BP1 recruitment DSBs and its regulation by phosphorylation. Recruitment of 53BP1 to DSBs involves recognition of the DSB by the MRN complex, ATM activation, and phosphorylation of histone H2A.X on residue Ser139 (γH2A.X). 53BP1 is phosphorylated on its 28 S/TQ sites by ATM. The ubiquitin ligases RNF8 and RNF168 are recruited downstream of ATM phosphorylation of yH2AX and are required for 53BP1 recruitment to chromatin. DYRK1A modulates the recruitment of 53BP1 to DSBs through interaction with RNF169. RNF169 competes with RNF168 for binding of 53BP1 and has a function in repair pathway choice by limiting 53BP1 at DSBs. Thus, DYRK1A enhances NHEJ by regulating the recruitment of RNF169 and 53BP1 to DSB sites. BRCA1 together with the DNA endonuclease CtIP trigger dephosphorylation of 53BP1, which repositions 53BP1 to the periphery and allows recruitment of HR factors such as BRCA1, Exo,1 and RPA to the center of the DDR focus, promoting HR (created by Biorender).

More recent evidence suggests that additional, non-canonical modes of 53BP1 recruitment to DSBs exist, involving regulatory functions of additional kinases. The dual-specificity tyrosine-regulated kinase 1a (DYRK1A) is a pleiotropic kinase, present in both the nucleus and cytoplasm, and its deregulation has been linked to neurological diseases (Altafaj et al., 2001; Aranda et al., 2011). DYRK1A modulates the recruitment of 53BP1 to DSBs through interaction with RNF169, a paralogue of RNF168. RNF169 competes with RNF168 for binding of 53BP1 and has a function in repair pathway choice by limiting 53BP1 at DSBs (Poulsen et al., 2012; An et al., 2018). Thus, DYRK1A enhances NHEJ by regulating the recruitment of RNF169 and 53BP1 to DSB sites (Figure 3). The dual specificity tyrosine phosphorylation-regulated kinase 2 (DYRK2) is also involved in DSB repair. In non-damage conditions, DYRK2 is mostly cytoplasmic and nuclear DYRK2 is constitutively ubiquitinated and degraded. In response to DNA damage, however, DYRK2 is phosphorylated by ATM, which prevents degradation and causes nuclear accumulation. Stabilized DYRK2 phosphorylates p53 at residue Ser46, suggesting that DYRK2 plays a role in p53 dependent apoptosis. The knockdown of DRYK2 impairs the formation of 53BP1 foci and HR efficacy (Yamamoto et al., 2017).

Interestingly, 53BP1 is an RNA-binding protein and may recognize DSBs via interaction of its tudor domain with damage-induced lncRNA (dilncRNA) in a Dicer-dependent manner. Indeed, transfection of antisense oligonucleotides specific for dilncRNA, mutation of the 53BP1 tudor domain, or depletion of Dicer, attenuates the formation of 53BP1 foci. Strikingly, 53BP1 foci formation in response to IR is sensitive to treatment with structure-specific RNases and the addition of RNA purified from damaged, but not non-damaged cells, rescues 53BP1 foci formation following RNase treatment (Pryde et al., 2005; Francia et al., 2012; Burger et al., 2017, 2019; Michelini et al., 2017; Botuyan et al., 2018). Thus, the efficient recruitment of 53BP1 to DSBs may involve the specific interaction with damage-induced RNA and its dependence on RNAi factors like Dicer suggests the involvement of dsRNA. Indeed, site-specific, DNA damage response RNA (DDRNA)/damage-induced (di)RNA accumulate in an RNAi factor-dependent manner in various organisms (Lee et al., 2009; Francia et al., 2012; Michalik et al., 2012; Wei et al., 2012). DDRNA/diRNA facilitate recruitment of a subset of secondary DDR factors, including 53BP1 and MDC1, to establish DSB foci and repair of DSBs, but are dispensable for the recruitment of primary DDR factors like the MRN complex (Francia et al., 2016).

Interestingly, additional DDR factors such as Rad51 and BRCA1 may also engage small ncRNA in an RNAi-like mechanism, where diRNA is complexed with the Argonaute family member Ago2 to guide the recruitment to DSBs. Using a DR-GFP/U2OS HR reporter system, the authors determine that Ago2 impairs HR comparably to RAD51 knockdown in mammals (Gao et al., 2014). Efficient recruitment of the acetyltransferase Tip60/KAT5 to DSBs also depends on small ncRNA (Wang and Goldstein, 2016). However, details on the structure of such transcripts are sparse and their physiological relevance remains controversial.

Furthermore, the tudor-interacting repair regulator (TIRR) was identified as binding partner of 53BP1 and regulator of DSB repair (Drané et al., 2017; Zhang et al., 2017). TIRR binds the tandem tudor domains of 53BP1, occupying the same binding site as H4K20me2. As binding of 53BP1 to TIRR occurs with ~25-fold higher affinity than binding to H4K20me2, TIRR outcompetes H4K20me2 for 53BP1 binding, thereby preventing 53BP1 recruitment to chromatin (Dai et al., 2018; Wang et al., 2018). 53BP1 is phosphorylated and released from the 53BP1-TIRR heterodimer in a RIF1- and ATM-dependent manner upon IR. Intriguingly, an additional, RNA-dependent mechanism of 53BP1-TIRR dissociation has been proposed. TIRR is an RNA-binding protein that interacts with a variety of transcripts (Avolio et al., 2018), and RNA molecules can displace the 53BP1-TIRR interaction *in vitro* (Botuyan et al., 2018). However, the molecular mechanism of RNA-mediated dissociation of the 53BP1-TIRR complex remains enigmatic.

The above examples illustrate the relevance of RNA for DSB recognition and suggest a complex regulatory network to engage DDR factors with the RNA. Future studies likely will extend a growing list of examples for the RNA-dependent DDR.

## Damage-Induced RNA Metabolic Enzymes in Response to UV Irradiation

Additionally to DSB repair, emerging evidence suggests that RNA metabolism plays a regulatory role in the repair of UV damage. Here, we review RNA-dependent DDR with focus on canonical and non-canonical responses to UV-induced DNA damage, highlighting novel, unexpected roles of RNAPII and phospho-isoforms of the RNAi machinery during nucleotide excision repair (NER).

Exposure to UVR triggers formation of DNA photo-adducts such as cyclobutane-pyrimidine dimers and is a natural driver of mutations. Two general pathways exist to recognize and repair UV-induced DNA damage in mammals: global genome (GG)- and transcription-coupled (TC)-NER (Marteijn et al., 2014). NER involves the formation of single-stranded DNA, potentially stalling of replication forks, and activation of ATM/ATR and downstream effector kinases, including Chk1 (Ciccia and Elledge, 2010). Critical factors for NER include Cockayne syndrome A/B (CSA/CSB), the UV-stimulated scaffold protein A (UVSSA), the ubiquitin-specific protease 7 (USP7), and Xeroderma pigmentosum factors A-F (XPA-XPF). During GG-NER, UV-induced signaling causes removal of RNAP II CTD phospho-marks and dynamic ubiquitination steps, which globally impairs both initiation and elongation of RNAPII transcription (Rockx et al., 2000; Sugasawa et al., 2005; Andrade-Lima et al., 2015). In TC-NER, the actively transcribing RNAPII machinery senses UV lesions and either stalls or performs trans-lesion RNA synthesis upon encountering DNA damage (Gregersen and Svejstrup, 2018). Thus, DDR signaling globally impairs RNAPII activity in response to UVR and triggers widespread ubiquitin-dependent proteasomal degradation, if TC-NER fails (Elia et al., 2015).

Interestingly, the comprehensive analysis of nascent RNA levels following UVR identified a subset of damage-induced transcripts such as ASCC3, which precede RNAPII inhibition and promote transcriptional recovery at DNA lesions (Williamson et al., 2017). In analogy to DSB signaling, the UV-induced DDR seems to be locally permissive to synthesize a subset of ncRNA transcripts with their production and/or processing being relevant for recognition and repair of UV lesions (Izhar et al., 2015) and transcription-initiation associated NER (Frit et al., 2002). Thus, a growing body of evidence suggests that the UV-induced DNA damage response modulates various different RNA metabolic processes, including transcription, splicing and translation (Munoz et al., 2009; Paronetto et al., 2011; Tresini et al., 2015; Wickramasinghe and Venkitaraman, 2016).

The MAPK effector p38 is an integral transducer of cellular stress and activated by numerous stress-inducing agents, including UVR (Brancho et al., 2003). Recent studies investigating the UV-induced phospho-proteome define p38 signaling as a critical regulator of the RNA metabolism after UV damage, in addition to ATM/ATR signaling (Borisova et al., 2018). In particular, p38 signaling preferentially targets RNA-binding proteins such as splicing factors, proteins involved in the turnover of AU-rich elements-containing mRNA, mRNA polyadenylation, and translation (Dean et al., 2004). The spectrum of targets is somewhat different from the UV-induced ATM/ATR phospho-proteome, which primarily identifies DNA-binding DDR factors. For example, p38-dependent phosphorylation of the negative elongation factor (NELF) complex promotes RNAPII elongation in a subset of genes upon UV damage. Phosphorylation of the NELF complex subunit NELF-E at residue Ser115 causes binding of the 14-3-3 proteins to NELF and its rapid release from chromatin (Borisova et al., 2018). The stimulation of RNAPII elongation occurs independently of the positive transcription elongation factor b (pTEFb), but is dependent on both CSB and XPC (Donnio et al., 2019). To reinitiate stalled RNAPII after completion of TC-NER the CTD phospho-mark Ser2 is reintroduced in a CSB-dependent manner. Reinitiation further involves the general RNAPII transcription factor H (TFIIH) and Cdk9 (Lainé and Egly, 2006; Anindya et al., 2010; Donnio et al., 2019). The serine-threonine kinase STK19 also promotes reinitiation of stalled RNAPII. Interestingly, STK19 mutations are critical drivers of melanoma (Yin et al., 2019). During TC-NER, STK19 interacts with CSB and accumulates at UV lesions and the depletion of STK19 causes hypersensitivity to UV damage. However, the precise molecular role of STK19 in TC-NER remains elusive. In addition, UV-induced DDR signaling can also target the RNAPII holoenzyme itself (Boeing et al., 2016). It will be important to elucidate further regulatory principles that control RNAPII activity in response to UV irradiation.

UV-induced DDR signaling is not limited to the control of RNAPII activity. In reminiscence to DSB signaling, the response to UV damage involves the RNAi factors Ago2, Dicer, Drosha and DGCR8 to control the DDR in both a miRNA-dependent and -independent manner. At the post-transcriptional level, UV damage causes an immediate-early relocalization phenotype of Ago2 into stress granules, which is accompanied by changes in the miRNA signature and altered expression levels of critical cell cycle regulators such as the Cdc25a phosphatase (Garinis et al., 2005; Pothof et al., 2009). The rapid, ATR-dependent degradation of Cdc25a upon UV damage (Mailand et al., 2000) is accompanied by induction of miRNA miR-16 to further destabilize Cdc25a transcripts by post-transcriptional gene silencing. The depletion of Ago2 or Dicer, in turn, impairs DDR signaling and cellular survival in response to UVR. Interestingly, Ago2 relocalization requires Cdk activity, but appears to be independent of ATM/ATR. However, the precise mechanism of UV-induced Ago2 relocalization remains elusive.

More recent evidence suggests an involvement of Dicer, Drosha, and DGCR8 in the UV-induced DDR besides post-transcriptional gene silencing. A subset of the cellular Dicer molecules accumulate in the nucleus to promote chromatin decondenzation in UV-irradiated cells (Chitale and Richly, 2017). Dicer chromatin occupancy depends on interaction with the transcriptional repressor ZRF1. The Dicer-dependent accumulation of the methyltransferase MMSET and dimethylation of histone H4 lysine 20 residues at UV lesions further stimulates NER and involves the scaffolding factor XPA. The individual depletion of Drosha or DGCR8 also results in hypersensitivity to UV irradiation (Calses et al., 2017). The importance of DGCR8 for the NER pathway is underscored by epistatic effects, which are caused by combining DGCR8 depletion with defects in XPA, CSA or CSB functions. The DDR signaling involves DGCR8 in NER by specific placement of the UV-induced DGCR8 phospho-residue Ser153. DGCR8 phosphorylation involves the MAPK effector JNK1a and confers resistance to UVR. With >20 mapped phospho-sites, DGCR8 phosphorylation is common and canonically involved in DGCR8 stabilization and enhancement of miRNA biogenesis (Herbert et al., 2013). Surprisingly, the function of Ser153 phosphorylated DGCR8 in NER is independent of its RNA-binding capability or interaction with its binding partner Drosha, and therefore likely miRNA-independent. Instead, phosphorylated DGCR8 physically interacts with RNAPII and CSB and does not alter miRNA biogenesis, indicating that phosphorylated DGCR8 promotes NER on chromatin in an RNA-independent manner. Concomitant with DGCR8 phosphorylation upon stress, the damage-induced phosphorylation of DGCR8, and its function independent of Drosha, represent some analogy to the involvement of phosphorylated Dicer in DSB repair (Yang et al., 2015). Such findings further underscore the crosstalk between the DDR and RNA metabolic factors.

Taken together, we described various regulatory principles that control the localization and activity of RNAPII and various RNAi factors in response to various DSB-induced phosphorylation events, underscoring the contribution of damage-induced transcripts for the recognition and repair of DSBs and the relevance of RNA-dependent DSB recognition.

## Concluding Remarks

Collectively, we have discussed the various interconnections of RNA metabolic enzymes with DNA damage-induced signaling, pointing toward an intimate crosstalk of a subset of DDR factors, including key regulatory proteins such as 53BP1, with RNA metabolism in response to both DSBs and UV lesions. Growing evidence indicates that some of the observed RNA-dependent DDR phenotypes may be generally employed by the DDR, while others seem to be locus-specific. With this in mind, a deeper understanding of Dicer functions in the DDR and its relevance for genome maintenance is of vital interest for cancer research.

Nevertheless, many questions remain in the emerging field of RNA-dependent DDR. How do RNAi factors discriminate transcripts at DNA lesions from canonical pri-/pre-miRNA substrates? Number of studies demonstrate the relevance of post-translational modifications for RNAi factors and their involvement in the DDR. It is tempting to speculate that complementary mechanisms exits that selectively direct phospho-isoforms to damage-induced transcripts rather than miRNA biogenesis. Interestingly, the epitranscriptomic mark N^6^-methlyadenosine (m6A) transiently and very rapidly accumulates at UV lesions to promote efficient DNA repair (Xiang et al., 2017). m6A is not required for the recruitment of canonical DDR factors such as XPA or TFIIH, but involves DNA polymerase kappa, indicating that m6A promotes translesion synthesis. Placement of m6A, potentially in combination with other marks, may also alter the conformation of transcripts and thereby create a DNA damage-specific eptitranscriptomic signature complementary to the damage-induced changes in posttranslational modification of both canonical DDR factors and non-canonical RNA metabolic enzymes involved in genome maintenance.

The engagement of RNA metabolic enzymes at DNA lesions creates a steric conflict between canonical DNA-binding DDR factors, which tend to protect DNA lesions from unscheduled activity of large multi-enzymatic complexes like the replisome or the RNAPII machinery and RNA metabolic factors which may even produce transcripts *de novo*. The understanding of spatio-temporal integration and regulatory principles of such seemingly counterintuitive processes will be a major advancement in the field.

## Author Contributions

KB, RK, and MG discussed the structure and content of the review. KB and RK wrote the first draft. MG and RK edited the draft.

### Conflict of Interest Statement

The authors declare that the research was conducted in the absence of any commercial or financial relationships that could be construed as a potential conflict of interest.
